# Gendered life courses and cognitive functioning in later life: the role of context-specific gender norms and lifetime employment

**DOI:** 10.1007/s10433-023-00751-4

**Published:** 2023-03-30

**Authors:** Ariane Bertogg, Anja K. Leist

**Affiliations:** 1 University of Konstanz, Constance, Germany; 2 Department of Social Sciences, University of Luxembourg, Esch-sur-Alzette, Luxembourg

**Keywords:** Cognitive ageing, Gender, Life course, Lifetime employment, Gender norms, Europe

## Abstract

With increasing life expectancy, dementia poses an epidemiological challenge. As a cure has not been developed, the investigation into preventive factors becomes pivotal. Previous research emphasizes the cognitively stimulating and socio-emotional benefits of lifetime employment, but research on heterogeneous patterns across social groups and societal contexts remains sparse. Sociological approaches have a promising potential to provide insights into health inequalities and can contribute to the study of this major societal challenge. We investigate the influence of previous employment biographies on cognitive functioning for men and women aged 50 to 75 in 19 European countries, using longitudinal and retrospective information from the Survey of Health, Ageing and Retirement in Europe. We link individual information on employment biographies and cognitive functioning to contextual measures of gender norms, using aggregated agreement rates to both men’s and women’s role in employment and family. We find that previous employment affects cognitive functioning men and women differently. Part-time employment is beneficial for women’s cognitive functioning, but not for men’s. Traditional gender norms are associated with lower levels of cognitive functioning for both genders and moderate the linkage between previous employment and cognitive functioning. In contexts with more traditional gender norms, men’s part-time employment is associated with lower and women’s part-time employment with higher cognitive functioning. We conclude that employment and non-employment participation can, depending on characteristics of individuals and contexts, benefit or hinder the life-course accumulation of cognitive reserve, and those with norm-deviating behaviour are disadvantaged.

**Supplementary Information:**

The online version contains supplementary material available at 10.1007/s10433-023-00751-4.

## Introduction

Cognitive ageing, particularly memory impairment, is the hallmark symptom of dementia (Albert 2011), a range of conditions typically arising in old age which co-occur with gradual decline of physical health (Livingston et al. 2020). Due to increasing numbers of people affected by dementia and the consequences for health and social care systems, dementia prevention has become a public health policy goal. Studies confirm the importance of lifetime employment for later-life cognitive functioning. Employment experiences over the life course, particularly *occupation and employment intensity* explain variation in older-age cognitive performance beyond education (Ford and Leist 2021; Finkel et al. 2009; Greenberg and Burgard 2021). Similarly, the contribution of childhood socioeconomic disadvantage on cognitive performance at older ages is mediated through both educational attainment and occupation (Aartsen et al. 2019; Ford et al. 2022).

Particularly occupational complexity, i.e. complex job tasks and skills, has been shown to be a valid proxy for cognitive stimulation and to influence cognitive trajectories (Andel et al. 2015; Finkel et al. 2009; Kröger, et al. 2008). Occupations also act through psychosocial work characteristics, such control over one’s tasks, on cognitive functioning (Nexø et al. 2016; Then et al. 2014). Cognitive stimulation has been put forwarded as a major determinant of cognitive reserve (Stern 2002), and *work-related cognitive stimulation* constitutes the major pathway through which lifetime employment influences cognitive functioning (Lövdén et al. 2020).

With regard to *employment intensity,* studies have concentrated on part-time vs. full-time employment. Part-time employment comes with better opportunities for work-care reconciliation and reduces stress. Moreover, from the literature on health and well-being benefits of part-time employment, one can assume that individuals also benefit cognitively from occupying *multiple roles* (Rozario et al. 2004). With regard to the role of lifetime part-time employment for cognitive functioning in later life, the evidence is less clear. Some studies find that, for mothers, part-time employment seems to be more beneficial than full-time employment; however, full-time employed mothers have higher cognitive functioning than non-employed mothers (Ice et al. 2020). Other studies (for instance, Roxo et al. 2021a) do not support such part-time benefits.

Additionally, homemaking and unpaid family caregiving can be cognitively enhancing. It has been argued that cognitive benefits from unpaid family caregiving—particularly parenthood—come from learning new tasks. Fewer studies have investigated the linkage between other types of non-employment (such as unemployment, illness) and cognitive functioning. It can be assumed that different situations leading to non-employment (e.g. parenthood, illness, unemployment) may also influence cognitive functioning at later ages (Leist et al. 2013). To sum up, various life course models provide different opportunities for cognitive reserve accumulation and cognitive stimulation via full-time employment, part-time employment, family caregiving and other unpaid activities.

Research indicates that biological and behavioural reasons for dementia are partly gender-specific (Artero et al. 2008; Ferretti et al. 2018). Some studies emphasize biological differences in brain ageing, e.g. due to stress (Snyder et al. 2016), or link biological differences in brain aging to possible roles of oestrogen and other sex hormones (Zarate et al. 2017). Yet, the role of these hormones is not an established finding, but an open research question that needs further attention (Snyder et al. 2016). With regard to behavioural factors, men and women show different health-promoting or risky behaviours, such as smoking, drinking, nutrition and exercise (Schünemann et al. 2017); however, associations between dementia-relevant risk factors and dementia are largely similar between the genders (Sindi et al. 2021; Geraets and Leist 2023). A promising way is to investigate life courses, as men and women differ considerably with regard to their participation in paid employment and unpaid activities, as well as family roles (Madero-Cabib and Fasang 2015; McMunn et al. 2015).

While a growing number of studies investigate how participation in employment relates differently to men’s and women’s cognitive functioning, fewer studies examine how these linkages depend on the macrosocial context (Engelhardt et al. 2009; Ice et al. 2020). Sociologists argue that consequences of biographies for later-life health are context-sensitive (Mayer 2018; Struffolino et al. 2016), as social contexts shape opportunities for the accumulation of health risks and protective resources (Ferraro and Shippee, 2009). More specifically, macrosocial contexts, with their gender cultures and welfare regimes, create opportunities for (gendered) accumulation of cognitive reserve both early in life (e.g. via education) and later in life (e.g. via gendered caregiving expectations and women’s labour market participation) (Weber et al. 2014; Wu et al. 2016). Comparative research shows that women’s employment biographies vary substantially across Europe (Van Winkle 2018). Studies evaluating context-level opportunities for accumulating cognitive resources refer to women’s access to education (Leist et al. 2021) and gender ideologies as drivers of gender gaps in cognitive functioning (Bonsang et al. 2017).

To sum up, while there is growing evidence of the importance of work intensity and family roles as micro-level mechanism to explain later-life cognitive functioning, and ample evidence on the role of gender cultures and gendered welfare regimes as macro-level influence on later-life health, few studies have addressed these two factors jointly, establishing micro–macro linkages needed to understand life course outcomes in their contexts (Mayer 2018). To study how different forms of previous (non-)employment affect later-life cognitive functioning according to gender cultures in macrosocial contexts is thus a highly promising way to understand this major challenge for ageing societies. We add to this emerging literature by investigating heterogeneity in the linkage between previous employment and cognitive functioning between men and women, and between societal contexts.

## Protective resources and risks factors for cognitive functioning in older age

Cognitive decline is a normal feature of aging (Salthouse 2010). As people age, their cognitive abilities start to decrease. “Cognitive reserve” refers to the set of cognitive skills and resources that are formed early in childhood and youth and accumulate across the life course (Stern 2002). Nevertheless, we observe substantial heterogeneity in cognitive functioning among the older population, both in terms of starting levels and speed of the decline trajectory. Cognitive functioning at older ages has been widely studied. Findings point to biological, behavioural, psychological, relational, social and macrosocial preventive and risk factors for cognitive decline. Biological factors include genetic risk and cardiovascular health, but also heightened levels of stress hormones, which has been shown to affect brain structures negatively (McEwen 2000). Behavioural factors comprise health behaviour, including smoking, nutrition, and exercise, and leisure activities which are cognitively stimulating, e.g. doing quizzes, puzzles or reading (Ihle et al. 2018). Psychological factors that may lead to faster cognitive decline include impaired mental health, most prominently depression, which has been shown to be a relevant risk factor for dementia (Livingston et al. 2020). Moreover, stress, which is often of psychological origin (Pearlin et al. 1981), also constitutes a risk factor for cognitive decline (Agbenyikey et al. 2015). Relational factors include social networks, regular contacts, volunteering and family relations, such as being partnered (Bertogg and Leist 2021) or looking after grandchildren (Arpino and Bordone 2014). Social factors include social class and living conditions, whereas macrosocial factors refer to the welfare context with its public health services and the cultural context with its normative ideals and prescriptions. These factors are not isolated but intersect across the life course. For instance, cardiovascular risk is the consequence of health behaviour, and changes in the family structure may trigger stress with cognitive consequences (Vidarsdottir et al. 2014).

## The role of employment and non-employment activities for cognitive functioning

To sum up, cognitive reserve is developed mainly earlier in life, while risk factors and resources *accumulate* over the entire life course. Employment is an important resource for cognitive functioning in later life. Its benefit emerges via two mechanisms. First, participation in the labour market provides opportunities for *cognitive stimulation.* This particularly applies to high-skill jobs, which are often characterized by higher degrees of occupational complexity and agency. Occupational complexity includes demanding work tasks and is linked to higher levels of cognitive functioning and lower risk of dementia (Andel et al. 2015; Finkel et al. 2009; Kröger, et al. 2008). Agency, i.e. a higher level of control over one’s workflows and tasks (Nexø et al. 2016; Then et al. 2014), is also associated with higher cognitive performance (Ford et al. 2021).

Second, studies document the *socio-emotional* benefits of being employed. Employed persons are exposed to regular social interactions and have larger a larger network of “weak” ties at their workplace (Berkman 2014; Granovetter 1983). Moreover, employment benefits well-being by providing a sense of purpose and community (Hinterlong et al. 2007; Rozario et al. 2004), which—in combination with family tasks—increase well-being.

Even though there is considerable institutional variation in Europe, shaping opportunities for labour market participation and the need for family-based care (Pfau-Effinger 2005; Saraceno and Keck 2010), men’s and women’s employment biographies differ substantially from each other within most countries, both in terms of quantity (i.e. duration and intensity) and quality (i.e. occupational complexity and agency) of lifetime exposure to employment (Madero-Cabib and Fasang 2015; McMunn et al. 2015). In the 19 countries studied in this article, men have spent on average over 90% of their life between 20 and 50 years in full-time employment, whereas full-time employment of women comprised only 66% of that time span. Moreover, among women, being a homemaker (10% of the time) or working part time (15% of the time) was much more common than among men (homemaking: 0.12%; part-time 2% of the time between ages 20 and 50).

Evidence suggests that the duration of exposure to (full-time) employment matters for later-life cognitive functioning (Greenberg and Burgard 2021), and that men and women may have similar returns (Ford and Leist 2021). The main mechanism brought forward in the literature refers to *cognitive stimulation* (Lövdén et al. 2020). Hence, it is plausible to assume that the more years someone has spent in full-time employment, the better their cognitive functioning in later life (H1). The benefits of full-time employment in terms of well-being and health are expected to extend to part-time employment as well. This is in line with role expansion theory (Thoits 1983; Nordenmark 2004), which argues that having *multiple roles* is beneficial, because multiple types of activities generate different resources (such as income, social network ties, satisfaction and a sense of purpose) that complement each other. However, having too many roles simultaneously or experiencing work-family reconciliation conflict may create *role overload* and generate stress which might negatively affect cognitive functioning (Sabbath et al. 2015; Chandola et al. 2006). Yet, the benefits from work-related *cognitive stimulation and multiple roles* should outweigh the potentially stressful effects of having to combine work and family. Being a (part-time) worker next to caregiving tasks or motherhood has been shown to be beneficial for well-being and health (Hinterlong et al. 2007; Rozario et al. 2004; Webber and Williams 2008). We thus assume that, previous part-time employment, similar to previous full-time employment, has beneficial effects on cognitive functioning later in life (H1).

Non-employment activities can be distinguished according to their cognitively enhancing potentials. Cognitive benefits may also emerge from non-employment activities which foster cognitive stimulation via *learning new skills*, or which foster complexity in everyday life. This includes being a homemaker and family caregiver. Particularly childbearing requires parents to acquire new skills and competencies during each phase of development of the child (Richards and Hatch 2011). Periods off work due to maternity have been shown to go along with lower cognitive decline in women as compared to periods off work without maternity, suggesting that a balance between work and family commitments can be beneficial for women (Leist et al. 2013). Even in the older birth cohorts studied in this article, most women have participated in the labour market before and after maternity. Hence, periods off work dedicated to homemaking are another way of combining employment careers and family roles across the life course. Similar to part-time workers, homemakers, too, may accumulate several social roles across phases with different paid and unpaid activities. Longer periods of time spent in homemaking may thus also be beneficial for cognitive functioning.

On the other hand, having been away from the labour market may hamper the chances of re-entry into the labour market, particularly into a position with higher complexity, autonomy and cognitive stimulation. An alternative argumentation postulates that longer spells spent in homemaking (if not combined with part-time employment) are associated with lower levels of cognitive functioning. To the best of our knowledge, these assumptions have not been tested yet. The finding that women who were exclusively homemakers exhibit stronger cognitive decline as compared to women who work full-time or combine work and care via part-time employment supports the latter idea (Leist et al. 2013). Overall, the second line of argumentation seems thus more plausible, motivating our second hypothesis assuming that longer time spent in homemaking should be associated with lower levels of cognitive functioning (H2).

*Non-productive time away from work* seems thus to pose the greatest risk of cognitive functioning (H3)*.* As one prominent example for non-productive non-employment, unemployment has been shown to be negatively associated with cognitive functioning, although here selection effects could play a role (Leist et al. 2013; Abrassart 2013). Retirement is another type of labour market non-participation. Evaluating the link between retirement and cognitive functioning is complex since selection into retirement needs to be accounted for. Accordingly, results are mixed (Bonsang et al*.,* 2017; Celidoni et al. 2017). Besides unemployment and retirement, there are still other types of non-employment, which include the inability to work due to ill health or disability, or being institutionalized, but also being in education, or travelling. Hence, there are a number of very heterogeneous non-productive inactivities, which may have differential effects on cognitive functioning (Leist et al. 2013). Few studies have investigated the distinct reasons for non-employment and their influence on cognitive reserve and cognitive functioning at later ages in detail. Because most of these activities occur quite rarely, a separate analysis of each of them is challenging and out of the focus of this study. We thus combine these different states into one single category, differentiating them only from unemployment.

We hence distinguish between five previous activities: full-time employment, part-time employment, homemaking, unemployment and other activities, for which we can formulate hypotheses. To sum up, we expect the following:

### H1

The longer the time an individual has spent in full-time and part-time employment between the ages of 20 and 50 years, the higher their levels of later-life cognitive functioning.

### H2

The longer the time an individual has spent in homemaking between the ages of 20 and 50 year the lower their levels of later-life cognitive functioning.

### H3

The longer the time an individual has spent in unemployment and other non-productive activities between the ages of 20 and 50 years, the lower their levels of cognitive functioning.

The general expectation is that paid employment, homemaking and the combination of both (e.g. in the case of part-time work) can all be cognitively enhancing the longer an individual has spent in these states. When compared relative to each other, the benefits of these three states may vary. Since it is yet not clear from the literature whether part-time employment carries the same cognitive benefits as full-time employment, or whether combining family tasks and work via part-time employment carries similar cognitive potentials as being a homemaker, we need to analyse these various aspects of previous (non-)employment biographies separately, hence also the separate hypotheses.

Moreover, these associations could be gender-specific, although the literature yields mixed findings on gender-specific employment-cognition relationships. Based on gender-role socialization, men and women exhibit different behaviours and fill in different social roles (West and Zimmerman 1987), and this also affects employment and family life courses (Moen 2001). The gender roles men and women are socialized with are shaped prevailing gender norms in the societal context. As has been shown for Europe, these norms vary not only between countries, but also within countries, across cohorts (Shorrocks, 2018). Moreover, women exhibit more egalitarian gender role attitudes than men in all countries studied. Men and women thus not only differ in their exposure to work-related cognitive stimulation and multiple role benefits; the benefits from these different activities may also be different for men and women. Particularly, having *multiple roles* may be more beneficial for women than for men. In most Western countries, even women in older birth cohorts have some formal education and training, preparing them for participating in the labour market. Yet, their cultural socialization has prepared them for being family caregiver, enabling them to adapt to both environments.

Men, in contrast, were less prepared to take on family care tasks in the context of their socialization and are still less likely to take on housework or retire early, even if they earn less than their wives (Hank and Jürges 2007; Bertogg et al. 2021). Some studies found that men are penalized on the labour market for being a family caregiver or for working part time (Coltrane et al. 2013; Fernandez-Lozano et al. 2020), which may offset some of the benefits stemming from multiple roles. Overall, one can thus assume that women, with their double socialization, may have larger benefits from reconciling employment and family via part-time employment. Not least, departing from less favourable economic positions than men, women’s cognitive gains via employment participation may be more pronounced than men’s. In order to shed light on gender-specific cognitive benefits of part-time employment, we analyse men and women separately and assume the following:

### H4

The beneficial effects of earlier part-time employment and homemaking are stronger for women than for men.

## Context-sensitive patterns of later-life health

Macrosocial characteristics have been shown to influence both levels and changes in cognitive functioning, as well as gender differences therein. Evidence stems from various research designs, which systematically examine how the context influences the accumulation of cognitive reserve, or shapes individuals’ exposure to protective and risk factors. Natural experiments regarding the length of compulsory schooling (Glymour et al. 2008; Schneeweis et al. 2014), and multilevel modelling testing the role of inequality of educational opportunity for men and women (Leist et al. 2021), have confirmed that the accumulation of cognitive reserve through early-life education depends on the opportunities provided by the context. Regarding the role of unemployment as a risk factor, studies have found that economic downturns—particularly during young adulthood, the phase in which cognitive reserve is accrued—influence cognitive functioning negatively (Leist et al. 2014).

A less explored source of macrosocial contextual influence lies in opportunities for work-related cognitive resource accumulation via cognitive stimulation. First studies have shown that strong welfare states reduce gender and class gaps in physical health in later life (Sieber et al. 2020; Uccheddu et al. 2019). Moreover, participation in various paid and unpaid activities was associated differently with cognitive development across eleven European countries (Engelhardt et al. 2009). These studies indicate that health gaps arising from (women’s) weaker economic positions are moderated by welfare institutions. It is often argued that cultural norms drive women’s and men’s different decision regarding labour market participation and multiple social roles (Pfau-Effinger 2005). Yet, with regard to cognitive functioning, no study has so far tested the role of gender norms for levels of cognitive functioning, or its moderating role of the cognitive benefits stemming from different employment-related experiences.

## Social norms and consequences of employment biographies

Traditional gender norms restrict women’s access to educational opportunities, which are crucial for building cognitive reserve (Roxo et al. 2021b; Weber et al. 2014). They also promote “standard models” of breadwinning and caring. Such standard models serve as frames of orientation for men’s and women’s family and labour market decisions and shape their employment biographies. Via these two mechanisms, gender norms may directly influence the accumulation of cognitive resources. Gender norms also work indirectly, by moderating the effects of life courses employment biographies on cognitive outcomes.

Social norms impose sanctions and “punish” individuals who deviate from standard models. Negative social sanctions often comprise social exclusion, stigmatization and reduced well-being. Compliance with norms, on the other hand, is “rewarded”, promoting gain in social status, integration and well-being (Elster 2009). As social integration and mental health are important resources for cognitive functioning, cognitive enhancement should depend on *how well one’s own life course fits with prevailing normative ideals*. Punishment for deviation may offset some of the benefits associated with work-related cognitive stimulation or occupying multiple roles, as negative social sanctions block social interactions needed for these benefits to emerge (Elster 2009). Conversely, reward may reinforce the benefits associated with employment or multiple roles. Such benefits are likely reinforced when individuals occupy high social status, interact frequently and high levels of well-being. Several studies show that the well-being and labour market consequences of caregiving differ according to the normative context (Verbakel 2014; Bertogg 2022). We assume that:

### H5 (“punishment”)

In contexts with stronger gender norms, individuals whose employment biography deviates from normative ideals (i.e. full-time working women, part-time working or homemaking men) exhibit lower levels of cognitive functioning than in contexts with weaker gender norms.

### H6 (“reward”)

In contexts with stronger gender norms, individuals whose employment biography conforms with the normative ideal (full-time employed men, part-time employed or homemaking women) exhibit higher levels of cognitive functioning than individuals whose employment biography does not conform with the normative ideal.

## Data and method

### Sample

This study draws on the Survey of Health, Ageing and Retirement in Europe (SHARE). We use waves 1–2 and 4–7 (collected between 2004 and 2019), as well as retrospective SHARELIFE interviews, which were collected first in wave 3 (2008, purely retrospective interview), or wave 7 (2017, mixed wave: retrospective and reduced panel for those respondents who did not take part in wave 3, and “normal” panel wave for those who did). Since not all countries ever sampled feature two full panel waves, we use data collected in 19 European countries (Austria, Belgium, Croatia, Czech Republic, Denmark, Estonia, France, Germany, Greece, Hungary, Italy, Luxembourg, the Netherlands, Poland, Portugal, Slovenia, Spain, Sweden and Switzerland).

SHARE waves 1 through 7 collected information from more than 119,000 participants living in a private home, who were observed in more than 300,000 person-year observations. We limit our analytical sample to those aged 50–75 years of age in order to avoid survivor bias and attrition selective on cognitive abilities in high old age. The selected age range includes 78% of the original sample. We exclude all respondents who have been diagnosed with dementia or brain cancer (see Ngandu et al. 2015), since we are interested in cognitive decline as an early hallmark for dementia and not progressed forms thereof (Albert 2011), or score high on depression scale (2 + SD above the mean), as depression impairs recall abilities. These three steps reduce our analytical sample to 96,466 persons. After excluding respondents who only took part in one panel wave (and hence, cannot be analysed longitudinally), we observe 74,519 participants in our sample 214,447 times (the eligible sample).

As our analyses rely on information regarding previous employment biographies, which were captured retrospectively in SHARELIFE interviews, we had to exclude all respondents for whom no SHARELIFE interview was available. In total, 72,173 only (about sixty per cent of the ever sampled respondents) took part in SHARELIFE and provided full information on previous employment. Among our analytical sample, 57,558 respondents (or 77% of the eligible sample for this study) answered the retrospective questionnaire, resulting in 180,509 valid person-year observations. Finally, we exclude all person-year observations with missing values on the dependent or explanatory variables (listwise deletion). This leaves us with 114,357 person-year observations nested in 43,860 respondents.

To sum up: from the overall SHARE sample, 62% are eligible due to age, health and panel participation. From the eligible sample (n = 74,519 persons), we retain 59% for analysis. A major part of lost cases (23%) can be attributed to unavailability of retrospective interviews, and around 19% being attributed to missing data on either the dependent variable (5%) or the explanatory variables (14%).

## Cognitive tests

A composite sum score from three cognitive tests serves as outcome variable. It measures three important dimensions of cognitive functioning, namely short-term and long-term memory, as well as executive functioning. These cognitive tests have been used for composite scores of cognitive functioning in different variations (Ford et al. 2021; Leist et al. 2021). Short-term and long-term memory consists of a ten-word learning list, which was captured with an immediate, respectively, delayed recall test. These scales range from 0 (no word remembered) to 10 (all ten words remembered). The third measure consists of a verbal fluency test, in which respondents were asked to name as many animals as they could in one minute (the observed values ranging between 0 and 100 animals). On average, respondents remembered 5.7 words at the immediate recall and 4.4 at the second, delayed recall, and named a total of 21 animals (SE 7.6). After standardizing the verbal fluency test to range from 0 to 10, by dividing the realized value by ten, the three scales were added up with a weight of 25% for each of the memory tests and 50% for the verbal fluency test. The final composite score ranges from 0 to 20. The average value on this composite score of cognitive functioning is 7.2 with a standard deviation of 2.1 (women’s mean: 7.4, men’s mean: 6.9). For our analyses, this score is z-transformed (yielding a mean of zero and a standard deviation of one); one unit corresponds to ten animals named or one word remembered.

## Previous employment biographies

In the SHARELIFE spell data, respondents provided information on their employment status for each year of life since completing education, or the age of fifteen, up to the interview. We recoded the original eighteen employment categories into five dichotomous variables, which—for each person and year of life—indicate whether the person has been in full-time employment (at least 31 working hours per week), part-time employment (max. 30 working hours per week), has been a homemaker, unemployed or “other”. “Other” combines all remaining non-employment spells, (such as disabled, travelling, volunteering, military service, being institutionalized or being education), which only occurred with low frequencies. The five dichotomous variables are mutually exclusive: individuals coded as being “full-time” employed at age 27 receive the value “0” on the other four variables. For each respondent, we aggregated these dichotomous variables over the age span from 20 to 50. This leaves us with five continuous variables ranging from 0 to 100, indicating the percentage of years between ages 20 and 50 spent in the respective state (see Figure A.1 in the supplementary files). Two retrospective control variables were computed the same way, namely: the percentage of time one lived with a partner (married or cohabiting) and the average number of children that one lived with during those 31 years.

## Gender norms

The measures for gender norms are derived from three rounds of the European Social Survey (ESS): round 2 (2004), round 4 (2010) and round 8 (2016). We aggregated the (weighted) percentage of *agreement* to the following two statements indicating two different aspect of gender norms: “Men should have more right to job than women when jobs are scarce” and “Women should be prepared to cut down on paid work for sake of family”. Higher agreement rates indicate a preference for a stricter gendered division of labour in the population. While this approach to measuring gender norms is quite novel in the analysis of later-life health, it has been used to explain retirement timing (Bertogg et al. 2021), well-being in the general population (Hagqvist 2016) and among informal caregivers (Verbakel 2014) and has been shown to contribute to explain gender gaps in cognitive functioning globally (Bonsang et al. 2017). Since gender role attitudes vary within countries—across time and birth cohorts (Inglehart 2002; Ebner et al. 2020)—we calculated the agreement rates separately for men and women from different birth cohorts in each country. Agreement ranges between 1 and 82 per cent (men have right to a job), respectively, 9 and 88 per cent (women should cut down) and vary considerably across birth cohorts countries (see Table A.2 and Figure A.5 in the supplementary files for the dispersion of values).

Gender norms, however, may also be associated with the economic well-being of a country (such as GDP per capita) or structural gender inequalities (such as women’s access to education and employment, life expectancy and political representation of women). In order to rule out that our findings are driven by the proposed normative mechanism of punishment and reward and not structural opportunities, we estimated robustness models adjusting for these variables. Women’s labour market participation or opportunities for full-time vs. part-time work may also depend on family policy characteristics. Hence, we additionally estimate models adjusting for country fixed effects.

## Covariates

To capture individual trajectories in cognitive functioning, the most important covariate in our models is the passing of time since the first measure (baseline). We created a time variable which takes on the value “0” at first observation and denotes the number of years since first observation for each subsequent observation. In order to allow for non-linear cognitive trajectories, we include a square term.

Further, we adjust for a number of important individual-level socio-demographic characteristics which influence both health risks and previous employment patterns. These variables include age at baseline as well as the square term thereof, in order to adjust for lower levels of cognitive functioning among those who entered the panel at later ages. We include information on birth cohort, which we use to match the norm variables to, with five categories: born before 1930, born 1930–1939, born 1940–1949, born 1950–1959 and born after 1959. Given that we control for cohort, age at baseline and time have to exclude period effects. Social stratification variables which affect both cognitive levels and employment trajectories are an individual’s highest educational level (ISCED 1 or 2, ISCED 3 or 4, ISCED 5 or 6, or not available). Another—subjective, but country-comparable—indicator for socio-economic status is the self-reported ability of the household to “make ends meet”. Finally, we control for whether one was born in the country (1 = yes).

As important correlates of impaired cognitive functioning and dementia, we control for physical health limitations (an index of mobility and functional health limitations) and depression, measured with the EURO-D scale. We control for whether one has received help or care due to health issues in the past 12 months.

Further control variables include information on respondents’ current life course situations and family roles at the time of the interview: we add current employment status (full-time, part-time, retired, inactive), civil status (married, repartnered, single, divorced, widowed) and the number of children and grandchildren (biological and non-biological, co-residing with the respondent or not), measured with two continuous variables. Representing family roles, we control for whether one has provided help or care to someone in the past 12 months.

Finally, we include five variables for cognitively stimulating job characteristics and behavioural factors which can mitigate cognitive decline. Occupational complexity is measured with two dichotomous variables: a high degree of autonomy and control at work, and the need to learn new things. These characteristics were asked for the current job among respondents still working and otherwise asked for the last job for those retired or not working. We further include the sector of concurrent or last employment by collapsing the 1-digit ISCO-88 of respondents’ concurrent or last occupation into four categories (agriculture, manufacturing, low-skill service and high-skill service). Cognitively stimulating activities are captured using two dummy variables: at least weekly participation in volunteering, associations, or clubs (“social activities”), and at least weekly doing puzzles or quizzes, or taking part in educational courses (“educational activities”).

## Analytical strategy

Observing respondents several times allows us to exploit the longitudinal variance both within and between individuals. We estimate Linear Random Effects Growth Curve models, with person-year-observations nested in persons. These models adjust for individual trends by including the time-variable with a random slope at the higher level. All models are estimated using Stata Version SE 16.1 using the *mixed* command. In the first step, we investigate the association between previous employment biographies and cognitive functioning using stepwise model building. In the second step, we enter separately the country-, cohort-, and time-specific gender norms variables. Finally, we investigate whether gender norms moderate the linkage between previous (non-)employment and cognitive functioning, by including interaction terms between these variables. All models are estimated separately for men and women.

## Results

### Employment biographies and cognitive functioning

**Fig. 1:**
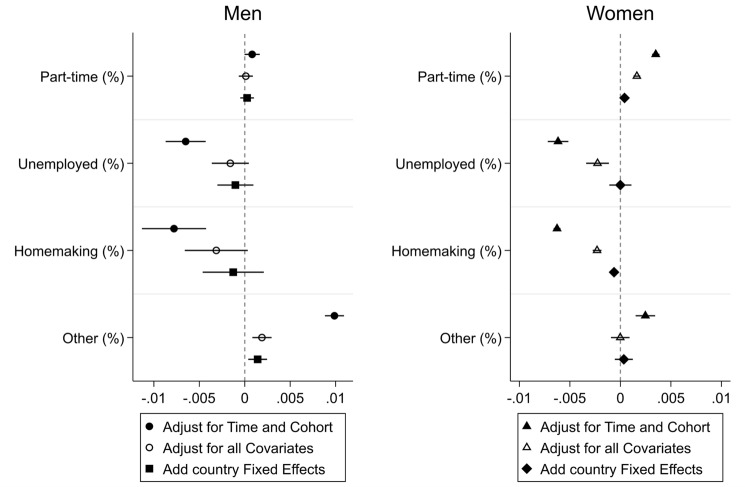
Effects of Duration of Previous (Non-)Employment on Cognitive Functioning Legend: SHARE, waves 1–2, 4–7, respondents aged 50–75 years without diagnosis of brain cancer or Alzheimer’s, observed at least twice, with valid information on the retrospective survey (wave 3 or 7). Multilevel Growth Curve models, stepwise model building. Model estimates in Table A.4 in the supplementary files. X-axis: Effect on z-standardized composite score (memory + verbal fluency, range (original metric): 0–20, 1SD = 6 scale points)

Figure 1 displays the association between durations of previous (non-)employment and cognitive functioning (Table 1 for coefficients). As the durations in all five states sum up to 100 per cent, we have to omit one of the variables, selecting full-time employment, as it is the most common state, which men (> 90%) and women (66%) have been in. When adjusting only for the time trend, age at baseline and birth cohort, we find that both men and women benefit from longer aggregate durations in part-time employment and in the “other” category (including higher education and training), supporting H1. Both genders have lower cognitive scores the more time they spent in unemployment or homemaking, supporting H2.

**Table 1:** Associations of Previous Employment Characteristics and Sociodemographic Covariates with Cognitive Score

	Women		Men	
	Full model	+ Country FE	Full model	+ Country FE
Part-time (%)	0.002^***^	0.000^*^	0.000	0.000
Unemployed (%)	− 0.002^***^	− 0.000	− 0.002	− 0.001
Homemaking (%)	− 0.002^***^	− 0.001^**^	− 0.003	− 0.001
Other (%)	− 0.000	0.000	0.002^***^	0.001^**^
Lives with partner (%)	0.083^***^	0.070^**^	0.164^***^	0.116^***^
Lives with children (%)	− 0.031^***^	− 0.033^***^	− 0.036^***^	− 0.030^***^
Time	0.032^***^	0.028^***^	0.034^***^	0.031^***^
Time, squared	− 0.003^***^	− 0.003^***^	− 0.004^***^	− 0.004^***^
*Birth cohort: before 1930 (ref.)*				
1930–1939	0.013	− 0.003	0.093	0.042
1940–1949	0.133	0.129	0.233^**^	0.158^*^
1959–1959	0.268^**^	0.291^**^	0.406^***^	0.327^***^
1960 or younger	0.397^***^	0.422^***^	0.590^***^	0.495^***^
Age at baseline	0.087^***^	0.088^***^	0.088^***^	0.089^***^
Age at baseline, squared	− 0.001^***^	− 0.001^***^	− 0.001^***^	− 0.001^***^
Number of functional limitations	− 0.024^***^	− 0.023^***^	− 0.026^***^	− 0.027^***^
*ISCED 1 or 2 (ref.)*				
3 or 4	0.377^***^	0.319^***^	0.322^***^	0.245^***^
5 or 6	0.649^***^	0.605^***^	0.569^***^	0.501^***^
Education: n.a	− 0.289^***^	− 0.173^***^	− 0.284^***^	− 0.200^***^
EURO-D depression scale	− 0.024^***^	− 0.024^***^	− 0.029^***^	− 0.030^***^
Vigorous weekly workout	0.042^***^	0.038^***^	0.049^***^	0.039^***^
Moderate weekly workout	0.084^***^	0.076^***^	0.073^***^	0.064^***^
*Current employment: Full time (ref.)*				
Part-time	− 0.009	− 0.012	− 0.013	− 0.007
Retired	− 0.060^***^	− 0.039^***^	− 0.072^***^	− 0.049^***^
Inactive	− 0.090^***^	− 0.062^***^	− 0.061^***^	− 0.047^***^
*Sector: Agriculture (ref.)*				
Manufacturing	0.100^***^	0.036	0.081^***^	0.063^***^
Service, low-skill	0.119^***^	0.063^**^	0.097^***^	0.096^***^
Service, high-skill	0.175^***^	0.109^***^	0.177^***^	0.172^***^
Social activities at least weekly	0.121^***^	0.098^***^	0.108^***^	0.084^***^
Educational activities at least weekly	0.092^***^	0.089^***^	0.061^***^	0.060^***^
*Married or cohabiting (ref.)*				
Repartnered	0.025	− 0.030	0.033	− 0.018
Single	0.008	− 0.004	− 0.123^***^	− 0.161^***^
Divorced	0.036^**^	− 0.003	− 0.003	− 0.041^**^
Widowed	− 0.029^*^	− 0.031^*^	− 0.049^*^	− 0.063^**^
Number of children	0.016^***^	0.018^***^	0.006	0.010^*^
Number of grandchildren	0.010^***^	0.003	0.006^**^	− 0.002
Given help or care to others	0.048^***^	0.041^***^	0.049^***^	0.037^***^
Received help or care from others	0.024^**^	0.006	0.010	− 0.004
*Household makes ends meet: easily (ref.)*				
With some/great difficulty	− 0.078^***^	− 0.043^***^	− 0.105^***^	− 0.068^***^
Information n.a	− 0.051^***^	− 0.040^***^	− 0.064^***^	− 0.054^***^
Born in country	0.134^***^	0.209^***^	0.129^***^	0.190^***^
Country fixed effects	No	Yes	No	Yes
n (person-years)	59,425	59,425	55,922	55,922

Source: SHARE, waves 1–2, 4–7, SHARELIFE. Respondents aged 50–75 years without diagnosis of brain cancer or Alzheimer’s, observed at least twice, with valid retrospective interview. Multilevel Growth Curve models

When including all other covariates, however, most of these results are no longer statistically significant at *p* < 0.05 for men, however, and effect sizes decrease for women. This indicates that the current situation, such as being employed, partnered, having children, mediates the effects of previous (non-)employment. When including country fixed effects to adjust for context-specific education and employment opportunities, women’s previous unemployment experiences are no longer statistically significant at *p* < 0.05.Thus, when adjusting for potential confounders and mediators, H1 and H2 only hold for women.

## Macrosocial differences in cognitive functioning

**Fig. 2:**
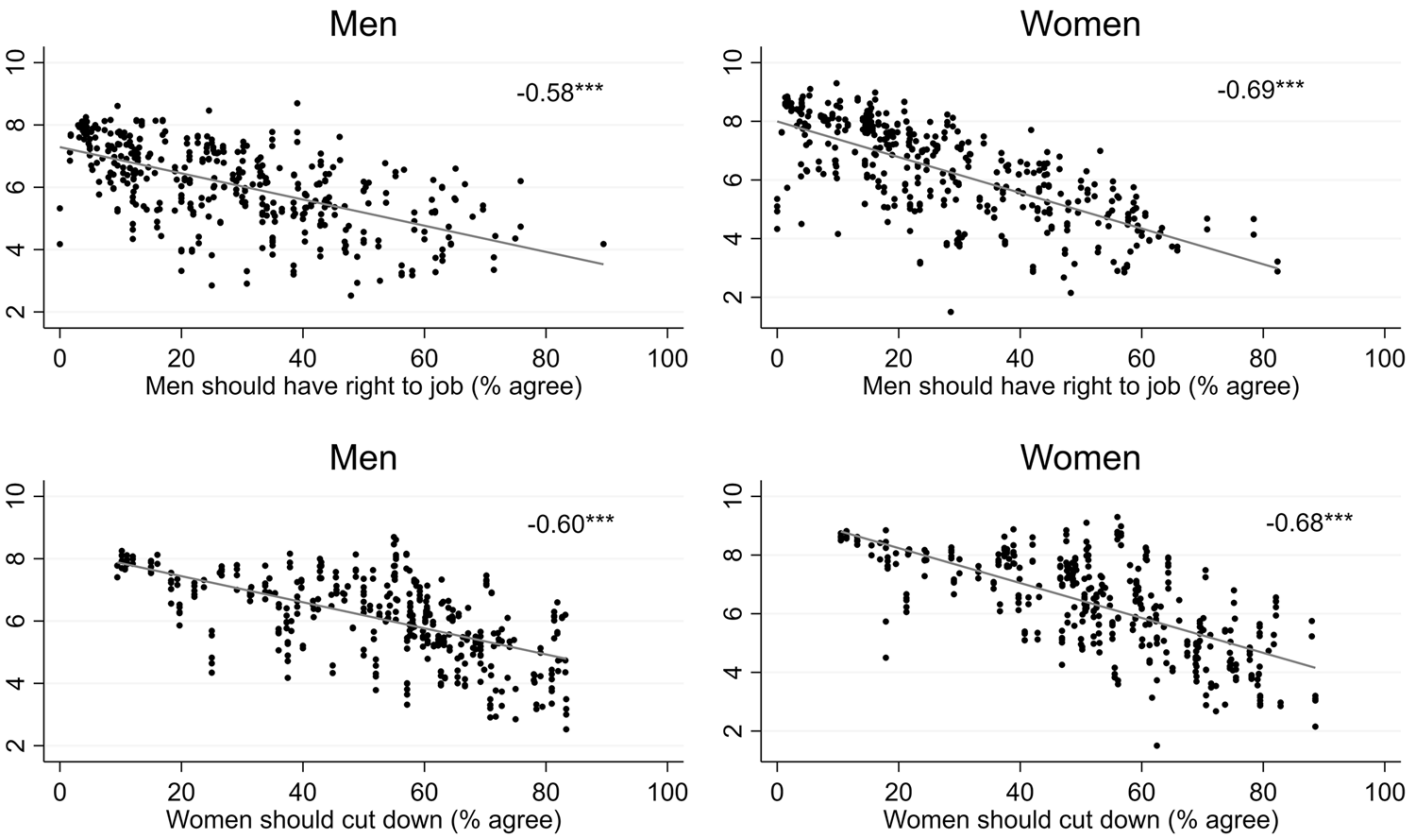
Bivariate Correlations of Cognitive Scores with Gender Norms Across Countries, Survey Years and Birth Cohorts Legend: Bivariate correlations, scatterplots with regression line. Y-axis: Average cognitive functioning scores by country, cohort and survey year (micro-level data: SHARE waves 1–2, 4–7, respondents aged 50–75 years without diagnosis of brain cancer or Alzheimer’s, observed at least twice, with valid information on the retrospective survey (wave 3 or 7)). X-axis: Gender norm indicators (data source: ESS rounds, 4, 5 and 8). Weighted aggregated agreement to two statements (1 = (fully) agree). over countries, birth cohorts and survey years, by gender. n = 816 gender-cohort-country-years. Correlation coefficients: Pearson’s r (two-tailed)

The next step turns to macrosocial differences. We assumed that gender norms both directly and indirectly affect cognitive potentials. As Fig. 2 indicates, individuals living in contexts with more traditional gender norms show lower levels of cognitive functioning. This association is highly significant and remains significant when adjusting for covariates and employment biographies (see Table A.4).

Finally, we investigate how gender norms moderate the association between employment biographies and cognitive functioning (Table 2). We assumed that the cognitive benefits of being exposed to work-related cognitive stimulation and multiple roles only apply to those men and women whose employment biographies conform to normative prescriptions (reward), whereas cognitive levels should be lower among those who deviate from the normative ideal (punishment). As expected (H4, punishment), we find that men’s longer time in part-time employment is associated with lower cognitive functioning in contexts with higher overall agreement to the norm that “men should have more right to a job than women”. Confirming our expectations (H5, reward), we find that women’s longer time in part time is associated with higher levels of cognitive functioning in contexts with higher overall agreement to the norm that “women should cut down on their jobs for the sake of the family”. Longer spells of unemployment or homemaking among women are negatively associated with cognitive functioning, and this effect is stronger the more traditional the gender norms. This is counter to what we had expected from H5. The highly significant “main effect” of gender norms reflects a general negative correlation for those who with previous full-time employment. For women, this could be expected (H4, punishment). For men, this finding is surprising; men do not seem to benefit from a protective effect of full-time employment when gender norms are strong.

**Table 2:** Interaction Terms between Gender Norms and Previous Employment – Associations with Cognitive Functioning

Gender norm indicator	Men		Women	
	Right to job	Cut down	Right to job	Cut down
Men should have right to job (% agree)	− 0.007^***^		− 0.011^***^	
Women should cut down (% agree)		− 0.091^***^		− 0.104^***^
% Part-time	− 0.000	− 0.000	0.001^**^	0.001^***^
Part-time * Men should have right to job	-0.000^**^		0.000	
Part-time * Women should cut down		− 0.001		0.001^***^
% Unemployed	− 0.002	− 0.002	-0.002^***^	− 0.002^***^
Unemployed * Men should have right to job	0.000		-0.000	
Unemployed * Women should cut down		0.000		− 0.001
% Homemaking	− 0.002	− 0.002	− 0.002^***^	− 0.001^*^
Homemaking * Men should have right to job	0.000		− 0.000	
Homemaking * Women should cut down		− 0.004		− 0.001^***^
Control variables	Yes	Yes	Yes	Yes
Random Slope (time)	Yes	Yes	Yes	Yes
n (person-years)	55,922	55,922	59,425	59,425
ICC (person)	0.477	0.479	0.468	0.474

Legend: SHARE waves 1–2, 4–7, respondents aged 50–75 years without diagnosis of brain cancer or Alzheimer’s, observed at least twice, with valid information on the retrospective survey (wave 3 or 7). Multilevel Growth Curve models, including all control variables. ICC = intra-class correlation. “Right to Job”: “If jobs are scarce, men should have more right to a job than women” (% agree, centred). “Cut Down”: “Women should prepare to cut down for the sake of the family” (% agree, centred). For robustness checks, see Table A.4

**Fig. 3:**
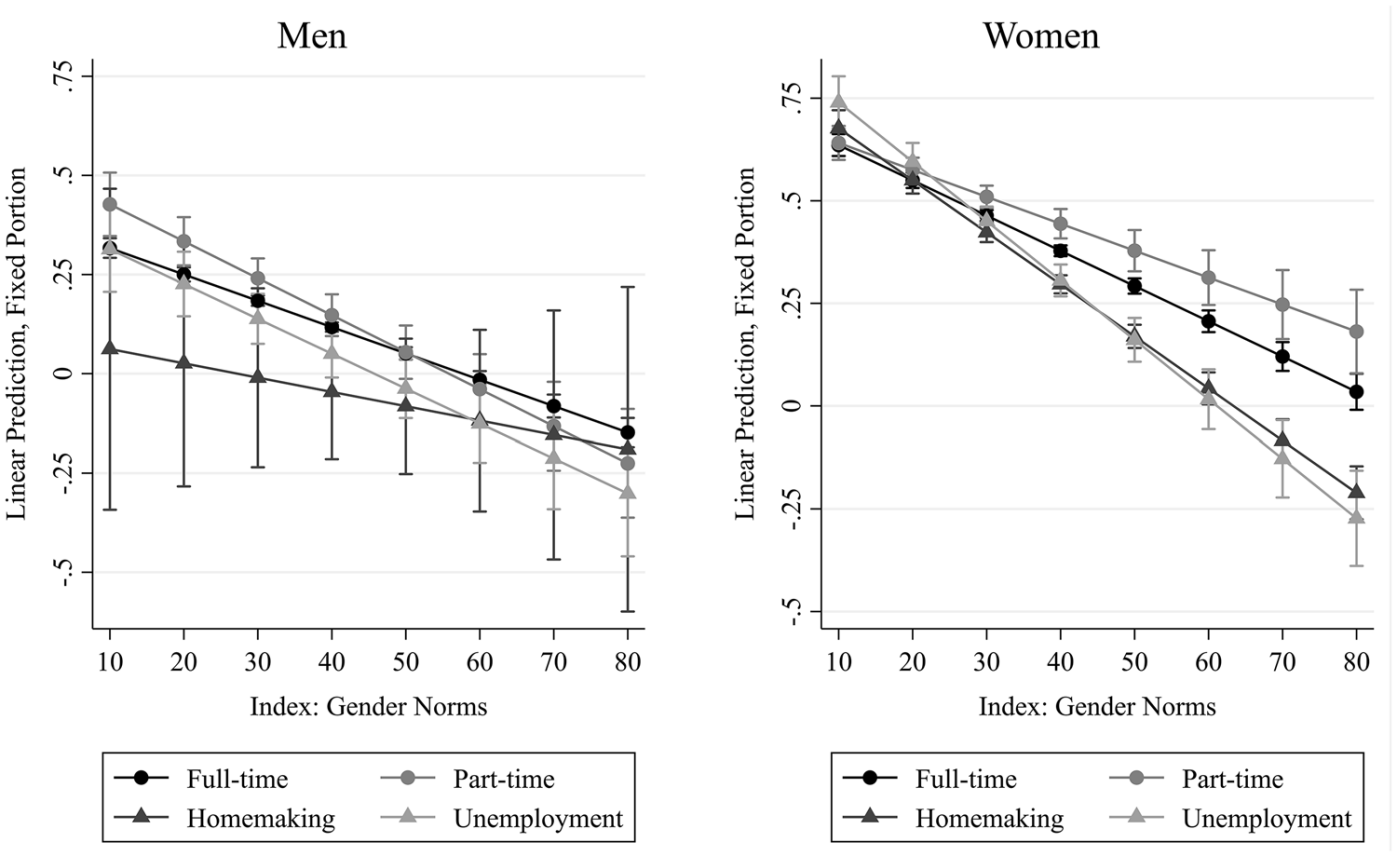
Association of Typical Trajectories with Cognitive Functioning Across Gender Norms Legend: SHARE waves 1–2, 4–7, respondents aged 50–75 years without diagnosis of brain cancer or Alzheimer’s, observed at least twice, with valid information on the retrospective survey (wave 3 or 7). Multilevel Growth Curve models with an interaction term between a gender norm index and typical trajectories. Gender Norm index: Both gender norms summed to an index ranging from 10–90 per cent agreement to either or both norms (Cronbach’s Alpha = 0.83). Typical trajectories = Gender-sensitive categorical variable (4 groups) indicating typical patterns of previous (non-)employment between ages 20 and 50: Full-time (Men min. 90% full-time employment, women min. 66% full-time employment), Part-time (Men > 0% part-time employment, women min. 10% part-time employment), Homemaking (Men > 0% homemaking, women min. 10% homemaking), Unemployment (Men min. 10% unemployment, women > 0% unemployment)

In order to interpret these findings in a more intuitive way, Fig. 3 shows predicted probabilities for four ideal–typical groups with various (non-)employment states. Groups were interacted with an index of both norm measures (see Figure A.1 and Table A.3 in the supplementary files). For all four typical groups, we find lower levels of cognitive functioning the more traditional the gender norm (downward pointing slopes). The degree to which stronger norms are associated with lower cognitive functioning depends on individuals’ biographies. Men have a slight benefit from having worked part time—but only in contexts where gender norms are egalitarian. In gender traditional contexts, they “lose” this advantage. Women whose biography comprised substantial part-time employment are not significantly different from women who predominantly worked full-time in contexts with egalitarian gender norms, but part-time becomes advantageous in contexts with more traditional gender norms.

In order to assess the robustness of our results, we included additional time-varying country-level variables regarding the economic situation (GDP per capita) and structural gender inequality (measured with the female employment rate among the 50- to 65-year-olds, and the Gender Inequality Index (GII)), and country fixed effects (separate models for each variable). GDP per capita and structural gender inequalities only partly mediate associations between gender and cognitive functioning for individuals with different labour market biographies. Thus, the cognitive benefits from egalitarian values work via another pathway than women’s educational and labour market or economic development. Such differences may be rooted in countries’ labour market policies, including social protection of part-time work (Nicolaisen et al. 2019). When controlling for country fixed effects, all interaction terms between previous employment and gender norms become insignificant. This suggests that heterogeneous patterns between individuals with different employment biographies may be explained by country-level factors.

## Discussion and conclusion

This article extends on previous research on the importance of life-course factors for later-life cognitive functioning by focusing on the role of previous employment biographies. It takes a gender- and context-sensitive approach to evaluate the potentials of cumulative exposure to five different forms of (non-)employment between the ages of 20 and 50 for later-life cognitive functioning. In doing so, this study contributes to both the literature on cognitive aging and sociological research on life-course influences on health. Another contribution lies in the theoretical linkage between sociological theory and an epidemiological perspective on ageing. More specifically, we argue that both employment and family caregiving may come with cognitive benefits, and work via two mechanisms: work-related cognitive stimulation and multiple-role benefits.

Our results support these suggested mechanisms. We find that longer spells of both full-time and part-time employment are beneficial for cognitive functioning, supporting the idea of work-related cognitive stimulation. For women, we find that part-time employment—as a means of reconciling family tasks and professional careers—comes with higher cognitive benefits, supporting the idea of multiple role enhancement and confirming previous findings documenting a positive effect of part-time employment on physical health and well-being (e.g. Rozario et al. 2004; Webber and Williams, 2007). Longer spells of homemaking are less beneficial for cognitive functioning, as are longer spells of unemployment.

Moreover, our findings document considerable contextual heterogeneity in how previous employment biographies affect later-life cognitive functioning. Extending on previous studies on women’s educational and employment opportunities, we focus on gender norms, measured as gender-, country-, cohort- and time-specific agreement to traditional male breadwinner and female caregiver roles. We find lower cognitive functioning among individuals in contexts where gender norms are more traditional. It can be assumed that a strict gendered division of labour blocks opportunities for experiencing multiple roles and hinders the accumulation of cognitive reserve for both genders. Furthermore, the association between previous part-time employment biographies and cognitive functioning is moderated by these norms. We find evidence for both *punishment* of deviation from traditional male breadwinner patterns, indicated by lower levels of cognitive functioning among men with previous part-time employment in gender traditional contexts, and *reward for compliance*, indicated by higher levels of cognitive functioning among women with longer spells of part-time employment in gender traditional contexts. These findings support our theoretical assumptions that negative social sanctions, such as exclusion and stigmatization, offset some of the cognitive benefits from part-time employment, while positive social sanctions, such as social integration and higher social status, reinforce these benefits.

Our findings have implications for policymaking. Enabling people to work and fostering reconciliation of paid employment and unpaid family care has been at the heart of social and family policy for decades. Yet, the long-term health consequences of lifetime work-and-care reconciliation are understudied. Our study documents the “long arm” of work- and family roles, as cognitive benefits or losses from previous employment accumulate up to advanced ages. Second, cognitive benefits seem gendered, with women being more susceptible to previous employment, while for men, the effects of previous employment biographies are largely absorbed by their current situation. This implies that for men, targeted interventions to improve cognitive functioning may ex post buffer the negative effects of earlier non-employment, for instance by addressing the current life situation and living conditions. For women, it should be more promising to promote the accumulation of cognitive benefits early in their careers. Women’s educational opportunities for building cognitive reserve have greatly improved in most Western countries in the last five decades (Weber et al. 2014), and many pension systems today aim at keeping women in the labour market for as much time as possible (Ebbinghaus and Hofäcker 2013). Policy designs promoting the reconciliation of family and career which are equally attractive for men and women, and social investment strategies to bring non-workers back into the labour market independent of their gender and family situation, have the potential to decrease cognitive risks and balance inequalities in cognitive decline.

This study comes with some limitations. First, while our measures of gender norms have a high external validity, linking individuals with the norms agree to among persons from the same birth cohort and country, we have no individual information on whether respondents embrace these norms. Even though theory argues that norms may influence individuals’ outcomes regardless of whether they embrace them (Elster 2009), individuals’ own attitudes could be a critical moderator; one we cannot test in this study. Second, the two explanations (punishment, reward) are documented in the sociological literature, but we lack information to determine which mechanisms are exactly at work (e.g. reduced well-being, social exclusion). Thus, our analysis remains one of ecological, macrosocial factors, rather than individual attitudes. Third, due to data availability, we have to rely on a recent measure of gender role attitudes and are not able to measure the attitudes held at the time when the men and women in our study were socialized or took work- and family-related decisions. Fourth, certain previous employment spells remain rare (e.g. volunteering), limiting the power to detect their cognitively enhancing or limiting potentials. Finally, it remains speculative to compare the societal and cultural significance of particular types of work and non-work across heterogeneous contexts. For instance, the degree to which part-time is a proxy for precarious employment with little social protection depends on the specific welfare context (Nicolaisen et al. 2019). Part-time employment is particularly prevalent and protected in some continental European welfare states with rather traditional gender norms, and relatively inclusive and stable economies, such as Austria, Switzerland, Germany and the Netherlands. Our robustness analyses confirm this interpretation.

To sum, norms on the gendered division of labour extend their influence beyond working age. Both men and women bear negative cognitive consequences if they live in contexts with traditional gender norms, and these are further exacerbated if their life courses deviate from these norms. Only women may to some extent benefit from traditional gender norms, namely when adopting a part-time employment model. Our study contributes to the emerging strand of literature which employs sociological concepts to study social influences on one of the major epidemiological challenges in ageing societies. With our research design linking a life-course perspective with macrosocial influence in the form of social norms, we are able to explain heterogeneity between and within contexts. The systematic analyses of gendered life courses and gender differences in the health consequences of gender norms point to important interactions between norms and life-course factors in maintaining cognitive health up to advanced ages.

## Supplementary Information

Below is the link to the electronic supplementary material.

Supplementary file1 (DOCX 3008 kb)
