# Supplementary material for: Gendered life courses and cognitive functioning in later life: the role of context-specific gender norms and lifetime employment
Source: Eur J Ageing. 2023 Mar 30;20(1):7. doi: 10.1007/s10433-023-00751-4 (PMC10063772; doi:10.1007/s10433-023-00751-4)
Supplement: Supplementary file 1 — Supplementary file1 (DOCX 3008 kb) [file 10433_2023_751_MOESM1_ESM.docx]

**Gendered Life Courses and Cognitive Functioning in Later Life: The Role of Context-Specific Gender Norms and Lifetime Employment**

**Supplementary Files**

**Table A.1: Sample Description**

|  | **Baseline** | **Total** | **Men** | **Women** |
| --- | --- | --- | --- | --- |
| Cognitive Score | 7.03 (2.05) | 7.22 (2.10) | 6.90 (2.04) | 7.54 (2.11) |
|  |  |  |  |  |
| Avg. number of person-year observations | 4.15 (1.43) | 4.76 (1.37) | 4.70 (1.40) | 4.81 (1.34) |
|  |  |  |  |  |
| *Trend, APC* |  |  |  |  |
| Time variable | 0 | 2.52 (2.31)  Min: 0  Max: 13 | 2.42 (2.30)  Min: 0  Max: 13 | 2.60 (2.31)  Min: 0  Max: 13 |
| Age at Baseline | 60.79 (7.12) | 60.85 (6.66) | 61.30 (6.57) | 60.41 (6.72) |
| Cohort: Born before 1930 |  |  |  |  |
| 1930-1939 |  |  |  |  |
| 1940-1949 |  |  |  |  |
| 1950-1959 |  |  |  |  |
| 1960 or later |  |  |  |  |
|  |  |  |  |  |
| *Previous Employment & Life Course* |  |  |  |  |
| % Full-time | 81.12 (28.96) | 79.47 (30.13) | 91.49 (15.67) | 67.90 (35.66) |
| % Part-time | 7.53 (21.23) | 8.63 (22.45) | 2.01 (11.51) | 15.00 (27.91) |
| % Homemaking | 4.74 (15.91) | 5.17 (16.47) | 0.12 (2.54) | 10.03 (21.86) |
| % Unemployment | 1.02 (6.51) | 1.03 (6.38) | 0.60 (4.29) | 1.44 (7.85) |
| % Other | 5.59 (9.44) | 5.70 (9.54) | 5.78 (9.08) | 5.63 (9.96) |
| % Partnered | 77.06 (25.19) | 77.53 (25.04) | 74.73 (24.43) | 80.21 (25.33) |
| % With children | 1.41 (0.88) | 1.42 (0.88) | 1.32 (0.84) | 1.52 (0.91) |
|  |  |  |  |  |
| *Covariates* |  |  |  |  |
| Full-time employed | 31.34 % | 23.87 % | 28.93 % | 19.00 % |
| Part-time employed | 14.42% | 14.24 % | 10.11 % | 18.20 % |
| Retired | 44.87 % | 52.76 % | 54.23 % | 51.35 % |
| Economically inactive | 9.38 % | 9.13 % | 6.73 % | 11.44 % |
| Sector: Agriculture | 7.66 % | 7.98 % | 8.39 % | 5.84 % |
| Industry / Manufacturing | 34.47 % | 32.79 % | 41.94 % | 2.96 % |
| Service, low skill | 22.13 % | 22.30 % | 22.85 % | 21.78 % |
| Service, high skill | 35.74 % | 37.82 % | 26.82 % | 48.39 % |
| Number of functional health limitations | 0.94 (1.77) | 1.14 (2.05) | 0.81 (1.88) | 1.36 (2.18) |
| Euro-D Depression Index | 1.53 (1.32) | 0.64 (1.14) | 0.58 (1.07) | 0.70 (1.20) |
| Married or Cohabiting | 77.27 % | 74.75 % | 81.82 % | 67.95 % |
| Repartnered | 3.03 % | 3.12 % | 3.29 % | 2.96 % |
| Single | 4.64 % | 4.69 % | 4.69 % | 4.69 % |
| Divorced | 8.57 % | 9.72 % | 7.04 % | 12.30 % |
| Widowed | 6.49 % | 7.72 % | 3.16 % | 12.10 % |
| Number of children | 2.09 (1.24) | 2.11 (1.24) | 2.22 (1.25) | 2.09 (1.22) |
| Number of grandchildren | 1.95 (2.49) | 2.24 (2.58) | 2.09 (2.52) | 2.38 ( 2.63) |
| Has given help or care | 33.51 % | 34.74 % | 32.79 % | 36.60 % |
| Has received help or care | 12.94 % | 15.76 % | 13.40 % | 18.02 % |
| Moderate activity at least weekly | 89.58 % | 88.80 % | 89.02 % | 88.58 % |
| Vigorous activity at least weekly | 58.92 % | 55.97 % | 59.16 % | 52.89 % |
| Takes part in social activity at least weekly | 36.66 % | 39.60 % | 40.30 % | 39.02 % |
| Takes part in educational activity at least weekly | 7.91 % | 16.72 % | 14.65 % | 39.00 % |
| Highest Educational Level: Not available | 2.45 % | 2.20 % | 2.44 % | 1.96 % |
| ISCED 1 or 2 | 30.03 % | 29.64 % | 29.55 % | 29.72 % |
| ISCED 3 or 4 | 41.26 % | 41.64 % | 42.45 % | 41.83 % |
| ISCED 5 or 6 | 26.26 % | 26.53 % | 26.56 % | 26.49 % |
| Household can make ends meet: (Fairly) Easily | 43.36 % | 49.66 % | 49.94 % | 44.49 % |
| With (some/large) difficulties | 24.78 % | 23.70 % | 21.06 % | 27.06 % |
| n.a. | 31.86 % | 26.64 % | 29.00 % | 28.45 % |
| Born in country | 91.53 % | 92.00 % | 92.08 % | 91.48 % |
| N (persons) | 40,318 | 40,318 | 21,037 | 19,281 |
| N (person years) |  | 130,732 | 63,846 | 66,886 |

**Table A.2: Country Characteristics**

|  | **N**  **(% sample)** | **Cognitive Score at Baseline**  **(Mean (SD))** | **Men right to job (% agree):**  **average** | **Women cut down (% agree):**  **average** | **Norm Index**  **(% agree) :**  **average** |
| --- | --- | --- | --- | --- | --- |
| Austria | 7901  5.97% | 7.73 (2.20) | 40.04 | 59.34 | 49.70 |
| Belgium | 12,601  9.52% | 7.31 (2.01) | 30.25 | 41.73 | 35.99 |
| Czech Republic | 11,713  8.85% | 7.02 (1.97) | 32.84 | 57.33 | 45.09 |
| Switzerland | 7,267  5.49% | 7.50 (1.89) | 27.59 | 68.45 | 48.02 |
| Denmark | 10,943  8.27% | 7.87 (1.72) | 9.03 | 22.01 | 15.51 |
| Germany | 10,544  7.97% | 7.50 (1.93) | 21.05 | 51.75 | 36.40 |
| Estonia | 10,030  7.58% | 7.43 (2.05) | 28.09 | 61.16 | 44.62 |
| Spain | 7,607  5.75% | 5.81 (1.97) | 27.45 | 63.35 | 45.40 |
| France | 10,040  7.59% | 6.84 (1.94) | 28.49 | 55.77 | 42.13 |
| Greece | 5,333  4,03% |  | 56.54 | 63.83 | 60.19 |
| Hungary | 958  0.72% | 6.78 (1.78) | 47.64 | 58.27 | 52.96 |
| Croatia | 1,738  1.31% | 6.89 (1.96) | 31.79 | 62.34 | 47.07 |
| Ireland | 384  0.29% | 6.95 (1.97) | 37.69 | 65.29 | 51.49 |
| Italy | 9,672  7.31% | 6.02 (1.89) | 62.19 | 75.11 | 68.65 |
| Netherlands | 3,845  2.91% | 7.00 (1.89) | 26.30 | 38.91 | 32.61 |
| Poland | 3,994  3.02% | 5.95 (1.85) | 38.93 | 66.33 | 52.63 |
| Portugal | 534  0.40% | 5.35 (1.98) | 33.93 | 62.88 | 48.40 |
| Slovenia | 5,878  4.44% | 6.94 (2.10) | 21.67 | 54.37 | 37.97 |
| Sweden | 9,711  7.34% | 7.60 (1.82) | 9.12 | 27.45 | 18.27 |
| n (country-gender-cohort-years) | 816 |  |  |  |  |
| n (person-years) | 130,732 |  |  |  |  |

Notes: Agreement to traditional gender role attitudes. Men right to job: “Men should have the right to a job if jobs are scarce”, captured in ESS rounds 2, 4, and 8. Women cut down: “Women should prepare to cut down for the sake of the family”, captured in ESS rounds 2 and 4. Dichotomized: 1=(Fully) Agree, 0=(Fully) Disagree. Weighted agreement rates by country, ESS round, gender, and birth cohort (same birth cohorts as Table A.1). Agreement rates from ESS round 2 (2004) matched to SHARE waves 1 and 2. Agreement rates from ESS round 4 (2010) matched to SHARE waves 4, 5 and 6 (as well as 7 and 8, cut down). Agreement rate from ESS round 8 (2016) matched to SHARE waves 7 and 8 (right to job). No indicator available for Luxembourg. Country-specific averages presented over both genders, all time points and cohorts (n=16 per country)

**Table A.3: Effect of Gender Norm Index (Basis for Figure 3)**

|  | (1) | (2) |
| --- | --- | --- |
|  | Women | Men |
| Gender Norm Index | -0.140^***^ | -0.103^***^ |
| Part-time (%) | 0.001^***^ | 0.000 |
| Gender Norm Index * Part-time (%) | 0.001^***^ | -0.001^*^ |
| Homemaking (%) | -0.002^***^ | -0.002 |
| Gender Norm Index * Homemaking (%) | -0.001^**^ | -0.002 |
| Unemployment (%) | -0.002^**^ | -0.002 |
| Gender Norm Index * Unemployment (%) | -0.001 | -0.000 |
| Other (%) | -0.000 | 0.002^***^ |
| Cohort: Born before 1930 (ref.) |  |  |
| 1930-1939 | -0.174^***^ | -0.371^***^ |
| 1940-1949 | -0.171^***^ | -0.278^***^ |
| 1950-1959 | -0.091^***^ | -0.150^***^ |
| 1960 or later | 0.000 | 0.000 |
| Age at baseline | 0.134^***^ | 0.096^***^ |
| Age at baseline, squared | -0.001^***^ | -0.001^***^ |
| Number of functional health limitations | -0.022^***^ | -0.025^***^ |
| Highest Educational Level: Not available | -0.342^***^ | -0.281^***^ |
| ISCED 1 or 2 (ref.) |  |  |
| ISCED 3 or 4 | 0.403^***^ | 0.339^***^ |
| ISCED 5 or 6 | 0.659^***^ | 0.585^***^ |
| EURO-D depression index | -0.019^***^ | -0.021^***^ |
| Vigorous activity at least weekly | 0.041^***^ | 0.044^***^ |
| Moderate activity at least weekly | 0.088^***^ | 0.073^***^ |
| Full-time employed (ref.) |  |  |
| Part-time employed | -0.013 | -0.026^*^ |
| Retired | -0.076^***^ | -0.086^***^ |
| Economically inactive | -0.108^***^ | -0.081^***^ |
| Takes part in social activity at least weekly | 0.107^***^ | 0.080^***^ |
| Takes part in educational activity at least weekly | 0.084^***^ | 0.083^***^ |
| Married or Cohabiting (ref.) |  |  |
| Repartnered | 0.020 | 0.046 |
| Single | -0.038 | -0.240^***^ |
| Divorced | 0.009 | -0.044^*^ |
| Widowed | -0.044^**^ | -0.089^**^ |
| Number of children | 0.005 | -0.004 |
| Number of grandchildren | 0.000 | 0.000 |
| Given help/care the past 12 months | 0.057^***^ | 0.047^***^ |
| Received help/care the past 12 months | 0.027^**^ | 0.020 |
| Household can make ends meet: n.a. | -0.030^**^ | -0.053^***^ |
| With (some/large) difficulties | -0.058^***^ | -0.074^***^ |
| (Fairly) Easily (ref.) |  |  |
| Born in country | 0.124^***^ | 0.168^***^ |
| Constant | -3.842^***^ | -2.951^***^ |
| *N(person-years)* | 47539 | 42473 |

**Table A.4: Robustness Analyses - Effects of Gender Norms: Raw, Adjusted, Interacted, Additional Controls and Country Fixed Effects**

|  | **Men** |  | **Women** |  |
| --- | --- | --- | --- | --- |
|  | Right to Job | Cut Down | Right to Job | Cut Down |
| *1) Raw Effect (only Norms and Time Trend)* |  |  |  |  |
| Right to Job | -0.013^***^ |  | -0.012^***^ |  |
| Cut Down |  | -0.244^***^ |  | -0.273^***^ |
| *2) Adjusted for Covariates* |  |  |  |  |
| Right to Job | -0.008^***^ |  | -0.012^***^ |  |
| Cut Down |  | -0.134^***^ |  | -0.137^***^ |
| *3) Adjusted for Covariates + Previous Employment* |  |  |  |  |
| Right to Job | -0.007^***^ |  | -0.011^***^ |  |
| Cut Down |  | -0.093^***^ |  | -0.102^***^ |
| *4) Interactions with Previous Employment, Adjusted for Covariates* |  |  |  |  |
| Right to Job (main effect) | -0.007^***^ |  | -0.011^***^ |  |
| * Part-time | -0.000^**^ |  | 0.000 |  |
| * Homemaking | 0.000 |  | -0.000 |  |
| * Unemployment | 0.000 |  | -0.000 |  |
| Cut Down (main effect) |  | -0.091^***^ |  | -0.104^***^ |
| * Part-time |  | -0.001 |  | 0.001^***^ |
| * Homemaking |  | -0.004 |  | -0.001^***^ |
| * Unemployment |  | 0.000 |  | -0.001^*^ |
| *5a) Interactions with Previous Employment, Adjusted for Covariates + GDP per capita* |  |  |  |  |
| Right to Job (main effect) | -0.006^***^ |  | -0.009^***^ |  |
| * Part-time | -0.000^**^ |  | 0.000 |  |
| * Homemaking | 0.000 |  | -0.000 |  |
| * Unemployment | 0.000 |  | -0.000 |  |
| Cut Down (main effect) |  | -0.071^***^ |  | -0.065^***^ |
| * Part-time |  | -0.001 |  | **0.000** |
| * Homemaking |  | -0.004 |  | -0.001^***^ |
| * Unemployment |  | 0.000 |  | **-0.001** |
| GDP per capita | 0.000^**^ | 0.000^***^ | 0.000^***^ | 0.000^***^ |
| *5b) Interactions with Previous Employment, Adjusted for Covariates + Female Employment Rate of Age 50-65* |  |  |  |  |
| Right to Job (main effect) | -0.002^***^ |  | -0.006^***^ |  |
| * Part-time | -0.000^**^ |  | 0.000 |  |
| * Homemaking | -0.000 |  | -0.000 |  |
| * Unemployment | 0.000 |  | -0.000 |  |
| Cut Down (main effect) |  | -0.001^*^ |  | 0.001^***^ |
| * Part-time |  | **-0.001^*^** |  | 0.001^***^ |
| * Homemaking |  | -0.004 |  | -0.001^**^ |
| * Unemployment |  | 0.001 |  | **-0.001** |
| Female Employment Rate | 0.009^***^ | 0.010^***^ | 0.010^***^ | 0.012^***^ |
| *5c) Interactions with Previous Employment, Adjusted for Covariates + Gender Inequality Index* |  |  |  |  |
| Right to Job (main effect) | -0.006^***^ |  | -0.010^***^ |  |
| * Part-time | -0.000^**^ |  | 0.000 |  |
| * Homemaking | 0.000 |  | -0.000 |  |
| * Unemployment | 0.000 |  | -0.000 |  |
| Cut Down (main effect) |  | -0.064^***^ |  | -0.066^***^ |
| * Part-time |  | -0.001 |  | 0.000^**^ |
| * Homemaking |  | -0.004 |  | -0.001^***^ |
| * Unemployment |  | 0.000 |  | **-0.001** |
| GII | 0.081 | -0.728^***^ | 0.255 | -0.961^***^ |
| *5d) Interactions with Previous Employment, Adjusted for Covariates + Country Fixed Effects* |  |  |  |  |
| Right to Job (main effect) | -0.009^***^ |  | -0.010^***^ |  |
| * Part-time | **-0.000** |  | -0.000 |  |
| * Homemaking | 0.000 |  | 0.000 |  |
| * Unemployment | 0.000 |  | 0.000 |  |
| Cut Down (main effect) |  | -0.139^***^ |  | -0.096^***^ |
| * Part-time |  | -0.001 |  | **-0.000** |
| * Homemaking |  | -0.003 |  | **-0.000** |
| * Unemployment |  | 0.001 |  | **-0.000** |
| Country Fixed Effects | Yes | Yes | Yes | yes |
| N (person-years) | 55925 | 55922 | 59428 | 59428 |

Note: **Bold coefficients** indicate a change towards the main model (model 4) interactions, all covariates) presented in the study.

**Table A.5: Effects of Gender Inequality Indicators, Interacted with Previous Employment**

|  | **Men** |  | **Women** |  |
| --- | --- | --- | --- | --- |
|  | Female employment ratio | Gender Inequality | Female employment ratio | Gender Inequality |
| Time | 0.033*** | 0.049*** | 0.043*** | 0.060*** |
| Time, squared | -0.005*** | -0.006*** | -0.006*** | -0.007*** |
| Female employment rate (50-65 year old) | 0.009*** |  | 0.011*** |  |
| Gender Inequality Index (GII) |  | -1.029*** |  | -0.127 |
| Part-time (%) | 0.000 | -0.001 | 0.000 | -0.001* |
| Part-time * Female employment | 0.000 |  | -0.000 |  |
| Part-time * GII |  | -0.018* |  | -0.024*** |
| Homemaking (%) | -0.003 | -0.003* | -0.002*** | -0.003*** |
| Homemaking * Female employment | -0.000 |  | 0.000 |  |
| Homemaking * GII |  | 0.028 |  | -0.028*** |
| Unemployment (%) | -0.001 | -0.001 | -0.001 | -0.003*** |
| Unemployment * Female employment | -0.000 |  | 0.000* |  |
| Unemployment * GII |  | 0.013 |  | -0.025* |
| Control variables included? | Yes | Yes | Yes | Yes |
| n (person-years) | 56847 | 56847 | 59156 | 59156 |

**Table A.6: Interactions between Individual-Level Variables**

|  | **Men** | **Women** | **Men** | **Women** | **Men** | **Women** |
| --- | --- | --- | --- | --- | --- | --- |
| Interaction | Cohort * Previous Employment | Cohort * Previous Employment | Norm Index * Time | Norm Index * Time | Current * Previous Employment | Current * Previous Employment |
| Norm Index | -0.103^***^ | -0.140^***^ | -0.092^***^ | -0.105^***^ | -0.103^***^ | -0.140^***^ |
| Cohort: Born <1930 (ref.) | 0.000 | 0.000 | 0.000 | 0.000 | 0.000 | 0.000 |
| 1930-1939 | -0.006 | -0.035 | 0.038 | -0.047 | 0.022 | -0.088 |
| 1940-1949 | 0.069 | -0.059 | 0.137 | -0.018 | 0.098 | -0.104 |
| 1950-1959 | 0.174^*^ | 0.021 | 0.266^***^ | 0.080 | 0.203^*^ | -0.034 |
| >1959 | 0.316^***^ | 0.108 | 0.416^***^ | 0.176 | 0.344^***^ | 0.046 |
| Part-time (%) | -0.034 | 0.004 | 0.000 | 0.000 | -0.000 | 0.000 |
| Born <1930 (ref.) * Part-time (%) | 0.000 | 0.000 |  |  |  |  |
| 1930-1939 * Part-time (%) | 0.034 | -0.004 |  |  |  |  |
| 1940-1949 * Part-time (%) | 0.034 | -0.003 |  |  |  |  |
| 1950-1959 * Part-time (%) | 0.034 | -0.003 |  |  |  |  |
| >1959 * Part-time (%) | 0.034 | -0.004 |  |  |  |  |
| Homemaking (%) | -0.008 | -0.002 | -0.002 | -0.002^***^ | -0.001 | -0.002^***^ |
| 1930-1939 * Homemaking (%) | -0.001 | 0.000 |  |  |  |  |
| 1940-1949 * Homemaking (%) | 0.007 | -0.000 |  |  |  |  |
| 1950-1959 * Homemaking (%) | 0.008 | -0.001 |  |  |  |  |
| >1959 * Homemaking (%) | -0.007 | -0.000 |  |  |  |  |
| Unemployment (%) | -0.094 | 0.003 | -0.001 | -0.002^***^ | -0.003 | -0.003^*^ |
| 1930-1939 * Unemployment (%) | 0.096 | -0.006 |  |  |  |  |
| 1940-1949 * Unemployment (%) | 0.092 | -0.005 |  |  |  |  |
| 1950-1959 * Unemployment (%) | 0.091 | -0.004 |  |  |  |  |
| >1959 * Unemployment (%) | 0.091 | -0.005 |  |  |  |  |
| Other (%) | 0.002^***^ | -0.000 | 0.002^***^ | -0.001 | 0.002^***^ | -0.000 |
| Norm Index * Time |  |  | -0.000 | -0.004^*^ |  |  |
| GDP per capita |  |  | 0.001^**^ | 0.002^***^ |  |  |
| Full-time (ref.) * Part-time (%) |  |  |  |  | 0.000 | 0.000 |
| Part-time * Part-time (%) |  |  |  |  | -0.000 | 0.001^*^ |
| Retired * Part-time (%) |  |  |  |  | 0.001 | 0.001^**^ |
| Inactive * Part-time (%) |  |  |  |  | -0.001 | 0.001^*^ |
| Full-time (ref.) * Homemaking (%) |  |  |  |  | 0.000 | 0.000 |
| Part-time * Homemaking (%) |  |  |  |  | 0.006 | 0.001 |
| Retired * Homemaking (%) |  |  |  |  | -0.005 | 0.000 |
| Inactive * Homemaking (%) |  |  |  |  | 0.001 | 0.000 |
| Full-time (ref.) * Unemployment (%) |  |  |  |  | 0.000 | 0.000 |
| Part-time * Unemployment (%) |  |  |  |  | 0.000 | -0.000 |
| Retired * Unemployment (%) |  |  |  |  | 0.002 | 0.002 |
| Inactive * Unemployment (%) |  |  |  |  | 0.001 | 0.001 |
| Constant | -3.200^***^ | -2.805^***^ | -3.359^***^ | -3.185^***^ | -3.219^***^ | -2.740^***^ |
| *N* | 55922 | 59425 | 49807 | 52539 | 55922 | 59425 |

**Table A.7: Separate Analyses for Memory and Verbal Fluency**

|  | **Men** |  |  |  | **Women** |  |  |  |
| --- | --- | --- | --- | --- | --- | --- | --- | --- |
| Dependent Variable | Memory |  | Verbal Fluency |  | Memory |  | Verbal Fluency |  |
| Previous Employment: Full-time (ref.) |  |  |  |  |  |  |  |  |
| Part-time | 0.000 | 0.000 | -0.000 | -0.000 | 0.001^***^ | 0.002^***^ | 0.000 | 0.001^*^ |
| Homemaking | -0.001 | -0.001 | -0.004 | -0.003 | -0.001^***^ | -0.001^***^ | -0.003^***^ | -0.004^***^ |
| Unemployed | -0.001 | -0.001 | -0.002 | -0.003^*^ | -0.001 | -0.001 | -0.004^***^ | -0.005^***^ |
| Other | 0.002^**^ | 0.002^**^ | 0.002^**^ | 0.001^*^ | 0.000 | 0.000 | -0.001^**^ | -0.002^***^ |
| Right to Job (main effect) | **-0.003^***^** |  | **-0.010^***^** |  | **-0.007^***^** |  | **-0.013^***^** |  |
| * Part-time | -0.000 |  | **-0.000^***^** |  | -0.000 |  | *0.000^*^* |  |
| * Homemaking | -0.000 |  | 0.000 |  | -0.000 |  | -0.000 |  |
| * Unemployment | 0.000 |  | -0.000 |  | 0.000 |  | *-0.000^**^* |  |
| Cut Down (main effect) |  | **-0.046^***^** |  | **-0.139^***^** |  | **-0.059^***^** |  | **-0.107^***^** |
| * Part-time |  | -0.000 |  | *-0.001^*^* |  | **0.001^***^** |  | 0.000 |
| * Homemaking |  | -0.003 |  | -0.003 |  | **-0.001^***^** |  | **-0.001^**^** |
| * Unemployment |  | 0.001 |  | -0.001 |  | -0.001 |  | **-0.001^*^** |
| Control Variables included | Yes | Yes | Yes | Yes | Yes | Yes | Yes | Yes |
| n | 56033 | 56033 | 56033 | 56033 | 59531 | 59531 | 59531 | 59531 |

Note: Memory is a sum score of immediate and delayed recall (range: 0-20). Verbal Fluency is the original scale of number of named animals. Both dependent variables are z-standardized. **Bold coefficients** are in line with the overall model using the composite score as dependent variable. *Coefficients in Italics* are not significant in the overall model.

**Figure A.1: Country, Cohort and Life Course Differences in Cognitive Score**

**
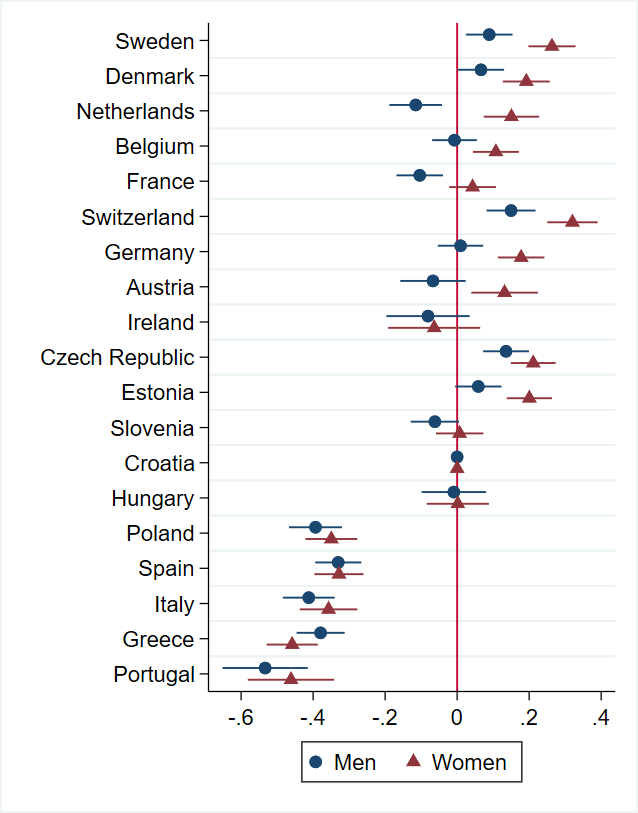
Figure A.2: Country Fixed Effects**

Legend: SHARE waves 1-2, 4-7, respondents aged 50-75 years without diagnosis of brain cancer or Alzheimer’s, and with at least 2 observations, as well as with valid information on the retrospective survey (wave 3 or 7). Multilevel Growth Curve models, coefficients for countries. X-axis: Effect on standardized composite score (memory + verbal fluency, range 0-20, 1SD = 6 scale points). Reference category: Croatia. Coefficients derived from Models 2 and 4 in Table 1.

**
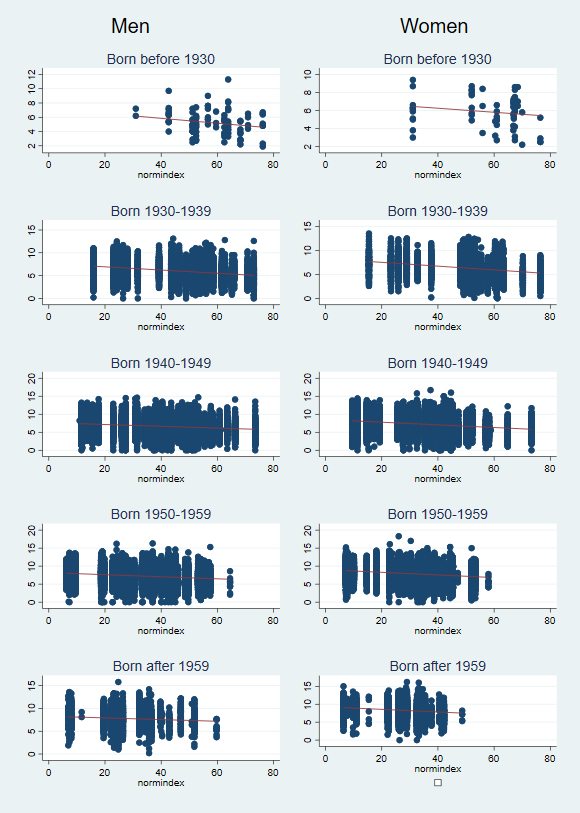
Figure A.3: Cohort-Specific Gender Norm Measures**

n=816 gender-cohort-country-years
